# Multiresponse Optimization of Inoculum Conditions for the Production of Amylases and Proteases by *Aspergillus awamori* in Solid-State Fermentation of Babassu Cake

**DOI:** 10.4061/2011/457392

**Published:** 2011-09-11

**Authors:** Aline Machado de Castro, Mariana Martins Pereira Teixeira, Daniele Fernandes Carvalho, Denise Maria Guimarães Freire, Leda dos Reis Castilho

**Affiliations:** ^1^Biotechnology Division, Research and Development Center, PETROBRAS. Avenide Horácio Macedo, 950. Ilha do Fundão, 21941-915 Rio de Janeiro, RJ, Brazil; ^2^COPPE, Chemical Engineering Program, Federal University of Rio de Janeiro, 21941-972 Rio de Janeiro, RJ, Brazil; ^3^Institute of Chemistry, Federal University of Rio de Janeiro, 21945-970 Rio de Janeiro, RJ, Brazil

## Abstract

This work aimed at investigating the simultaneous production of amylases and proteases by solid-state fermentation (SSF) of babassu cake using *Aspergillus awamori* IOC-3914. By means of experimental design techniques and the desirability function, optimum inoculum conditions (C/N ratio of propagation medium, inoculum age, and concentration of inoculum added to SSF medium) for the production of both groups of enzymes were found to be 25.8, 28.4 h, and 9.1 mg g^−1^, respectively. Significant influence of both initial C/N ratio and inoculum concentration was observed. Optimum amylolytic activities predicted by this multiresponse analysis were validated by independent experiments, thus indicating the efficacy of this approach.

## 1. Introduction

Amylases comprise a group of hydrolases used in the breakdown of starchy homopolysaccharides, that is, amylose, a linear *α*-1,4-linked glucose-based polymer, and amylopectin, a glucose-based polymer with linear chains and *α*-1,6-linked branches [1]. In the production of ethanol, two major groups of amylases are important [2–4]: endoamylases (liquefying enzymes), which are composed mainly of *α*-amylases (EC 3.2.1.1) and release oligosaccharides of various lengths by the random attack of internal *α*-1,4 linkages and exoamylases (saccharifying enzymes), composed mostly by glucoamylases (EC 3.2.1.3), which release glucose as the main product by cleaving terminal *α*-1,4 bonds. 

In previous works, alternative feedstocks, such as babassu (*Orbygnia phalerata*) [5] and castor bean (*Ricinus communis*) [6] were considered for the production of ethanol in Brazil due to their abundance. However, these materials, in addition to starch, contain considerable amounts of other fractions (proteins and other polysaccharides) [5, 6], which hinder the exposure of starch to amylases. An approach that has been proposed by other authors to improve ethanol yield consists of using proteases (exopeptidases, EC 3.4.11.x–3.4.18.x, and endopeptidases, EC 3.4.21.x–3.4.25.x) in combination with amylases [7, 8].

For the production of several bioproducts, especially enzymes, solid-state fermentation (SSF) processes are preferred over submerged fermentation (SmF) due to a number of advantages, including higher product concentration, lower catabolite repression [9], and lower operational costs [10, 11]. However, SSF is not the most widely used large-scale fermentation technology, and this can be attributed to engineering bottlenecks for its scaleup [11]. The challenges include difficulties in bioreactor design to avoid undesirable effects of overheating [12] and limitations related to inoculum propagation, particularly in the case of fungal processes based on inoculum using spores [13].

The impact of spore generation on the economic aspects of SSF processes was previously studied by de Castro et al. [14], who compared five culture media for inoculum propagation and observed up to 7.5-fold cost differences to generate the amount of spores (10^10^) required to inoculate a large-scale process. Moreover, Gutarra et al. [13] searched for new alternatives to spore inoculum, including the use of fungal pellets propagated by SmF, as well as the use of fermented solids obtained by small-scale SSF employing the same feedstock as in the main fermentation. Generally, the larger the scale, the more important the selection of a proper inoculum propagation strategy for an SSF process. Therefore, considering the production of amylases, which major application is in the hydrolysis of starch for the production of a biofuel, the optimization of inoculum propagation conditions is of paramount importance in the process economics. 

Different authors have used statistical designs to optimize amylases production. However, the focus was largely on fermentation conditions [15–17]. From the several works that investigated culture media composition for the production of amylases [17–27], the influence of carbon and nitrogen sources became evident, and malt extract and peptone were pointed out as some of the nutrients which enhanced the best amylases production. Thus, the use of these compounds also in inoculum propagation steps could preadapt cells and so decrease lag time in the main fermentation and increase enzyme productivity.

Thus, in this work, the use of different inocula obtained by SmF as well as the optimization of inoculum propagation conditions were investigated. Inoculum age and concentration, and the C/N ratio of inoculum propagation media composed solely by malt extract and/or peptone, were evaluated in order to maximize the simultaneous production of amylases and proteases by *Aspergillus awamori*, using as raw material an agroindustrial byproduct largely available in Brazil (babassu cake). To our knowledge, this is the first report on the multiresponse analysis of the production of amylases and proteases using this feedstock.

## 2. Experimental

### 2.1. Raw Material

Babassu cake (mean particle size of 923 *μ*m), which is an agroindustrial byproduct generated during the extraction of oil from babassu seeds, was kindly provided by TOBASA Bioindustrial de Babaçu S.A. (Tocantinópolis, Brazil). For the solid-state fermentation studies, the cake was dried, ground, and sieved to obtain particles in the range of 210 to 297 *μ*m (65 and 48 mesh Tyler, respectively).

### 2.2. Microorganism Maintenance

*A. awamori* IOC-3914 was obtained from Instituto Oswaldo Cruz (IOC) culture collection. Before propagation in the liquid propagation media, cells were kept for 7 days at 30°C in starch agar medium, as described by Castro et al. [14].

### 2.3. Inoculum Propagation in Liquid Media

*A. awamori* was grown in different conditions in SmF at 30°C and 200 rpm in 250-mL shaken flasks containing 100 mL of culture medium. Propagation time (inoculum age), C/N ratio of the medium, and inoculum concentration were studied at the levels shown in Table 1 according to a central composite rotatable design (CCRD). The influence of the C/N ratio of the medium was evaluated by combining different proportions of peptone and malt extract but maintaining the sum of both concentrations fixed at 35 g L^−1^. Thus, the culture medium with the lowest C/N ratio (3.0) contained only peptone as carbon source, whereas the medium with the highest one (C/N = 25.8) contained only malt extract as nutrient. The central points (CP) of the experimental matrix were carried out in quadruplicate.

The growth kinetics of the fungus in each of the culture media with the C/N ratios shown in Table 1 was studied for each inoculum condition. With this purpose, dry biomass was determined after filtration through 0.22 *μ*m membranes and drying until constant mass. These biomass data were adjusted according to a logistic model for microbial growth (1) [28]

(1) $$\begin{array}{c} X=\frac{X_{m}}{1+\left(\left(X_{m}/X_{0}\right)-1\right)\times e^{-\mu t}} \end{array},$$

where *X*, *X*_0_, and *X*_*m*_ represent biomass concentration at a propagation time *t*, biomass concentration upon inoculation and the predicted maximum biomass concentration, respectively. Equation (1) is also useful for predicting the specific growth rate (*μ*) and, as consequence, the doubling time (*t*_*d*_) of the cells.

### 2.4. SSF Experiments

*A. awamori* cells propagated under different conditions were inoculated at different concentrations in lab-scale tray bioreactors containing 2.5 g of babassu cake, according to Table 1. The initial moisture content of all experiments was adjusted to 70%. The trays were incubated for up to 120 h at 30°C. Regularly, whole trays were taken as samples and submitted to enzyme extraction, as previously described [14]. 

For the final validation of results under the optimized conditions, in addition to the standard scale, an experiment in a 4-fold larger scale was performed, that is, with 10 g of babassu cake. All conditions were kept the same as in the standard fermentations, including bed characteristics (e.g., mass to area ratio equal to 2.46 kg_cake_  m^−2^_tray_).

### 2.5. Experimental Analyses

C/N ratio of malt extract and peptone was calculated based on analyses (in duplicate) on an Elemental Analyzer 2400 CNH (Perkin Elmer). 

Microbial biomass samples collected after growth in different liquid media were coated with gold and observed using a scanning electron microscope (model INCAPentaFETx3, Oxford Instruments, Oxford, UK). The accelerating voltage was set to 20 kV for all images.

Regarding enzyme assays, endoamylase, exoamylase, and proteases activities were determined using 0.5% (m/v) soluble starch, 1.0% (m/v) soluble starch, and 0.5% (m/v) azocasein (Sigma Aldrich, St Louis, USA) as substrates, respectively, as described by de Castro et al. [29]. In all cases, results are expressed as mean 1± standard deviation (SD).

### 2.6. Statistical Analyses

Amylolytic and proteolytic activities detected in the SSF experiments were analyzed using the software Statistica 8.0 (Statsoft Inc, Tulsa, OK, USA). After analysis of variance (ANOVA) and normality test of the results, nonlinear regression was performed to obtain models to predict the responses, as shown in (2). In this equation, *Y* represents the dependent variables or responses (*k* = 3: endoamylase, exoamylase, and proteases activities); *ξ*_0_, *ξ*_*i*_, *ξ*_*ii*_, and *ξ*_*ij*_ represent the regression coefficients for the central, linear, quadratic, and interaction terms of the model, respectively, and *X*_*i*_ and *X*_*j*_ represent the independent variables or factors (1: C/N ratio, 2: inoculum age, and 3: inoculum concentration) in terms of their original (nonnormalized) levels

(2) $$\begin{array}{c} Y_{k}=\xi_{0}+\overset{n}{\underset{i=1}{\sum }}\xi_{i}X_{i}+\overset{n}{\underset{i=1}{\sum }}\xi_{ii}X_{i}^{2}+\overset{n}{\underset{i,j=1}{\sum }}\xi_{ij}X_{i}X_{j} \end{array}.$$

After determining the models for the three responses, their combined behavior as a function of the different inoculum conditions was analyzed using the global desirability function [30] as the objective function. For both the simultaneous analysis of exoamylase and endoamylase production (*k* = 2), and the simultaneous analysis of the production of the three groups of enzymes (*k* = 3), (3) was adopted. In this equation, *D* represents the global desirability value and *d*_*k*_ stands for the individual desirability values of the responses, calculated as the ratio of the response under given conditions to the maximum value obtained for that response considering the whole experimental space investigated

(3) $$D={\left(\overset{k}{\underset{i=1}{\prod }}d_{k}\right)}^{1/k}.$$

## 3. Results and Discussion

### 3.1. Inoculum Propagation of A. awamori in Liquid Media

After initial spore propagation in starch agar medium, *A. awamori* was grown in a liquid propagation medium containing malt extract and peptone at a C/N ratio of 22 in order to investigate growth kinetics and to establish the levels of inoculum age to be evaluated in the CCRD experiments. According to the growth curve obtained (data not shown), at 11.2 h the cells were in the acceleration phase, at 16, 23, and 30 h they were in the beginning, middle, and end of the exponential phase, respectively, whereas at 34.8 h, cells were in stationary phase.

*A. awamori* was then cultivated in culture media with different C/N ratios (Table 1), and the growth curves for each condition were determined. It was observed that within the ranges evaluated, the higher the C/N ratio, the faster the kinetics, evidenced by the specific growth rate (*μ*) and the doubling time (*t*_*d*_), as shown in Table 2. The specific growth rates observed (0.02–0.15 h^−1^) were lower than those reported by Hellendoorn et al. [31] (0.28–0.40 h^−1^) for cultivation of *A. awamori* in an airlift reactor, and this could be possibly due to oxygen transfer limitations in the shaken-flask cultures.

For the CCRD experiments, the dry weights of *A. awamori* cells after growth in the different media for different propagation times were at first determined (Table 3), in order to calculate the necessary amount of cell suspension to inoculate in babassu cake so as to meet the levels of inoculum concentration previously established (Table 1). Aiming at to simplify and integrate steps and to decrease costs when the process is scaledup, the liquid media from the submerged inoculum propagation stage (containing residual nutrients) were completely and solely used for moisture adjustment in the SSF. In the experiments where higher C/N ratios and inoculum ages were adopted, the culture supernatants were more viscous, possibly due the production of exopolysaccharides. According to Barbosa et al. [32], such condition favors the production of these molecules.

In the liquid propagation media, cells grew as mycelial biomass. Hyphae diameters were in the range of 1.8–3.4 *μ*m, but in some cases, larger hyphae measuring 10.5–11.5 *μ*m were observed. The hyphal organization pattern and absence of asexual reproductive structures detected in the present work are similar to those observed by Gutarra et al. [33] when *Penicillium simplicissimum* was propagated in a semisynthetic liquid medium.

### 3.2. Experimental Results of Amylases and Proteases Production by SSF

**Figure 1:**
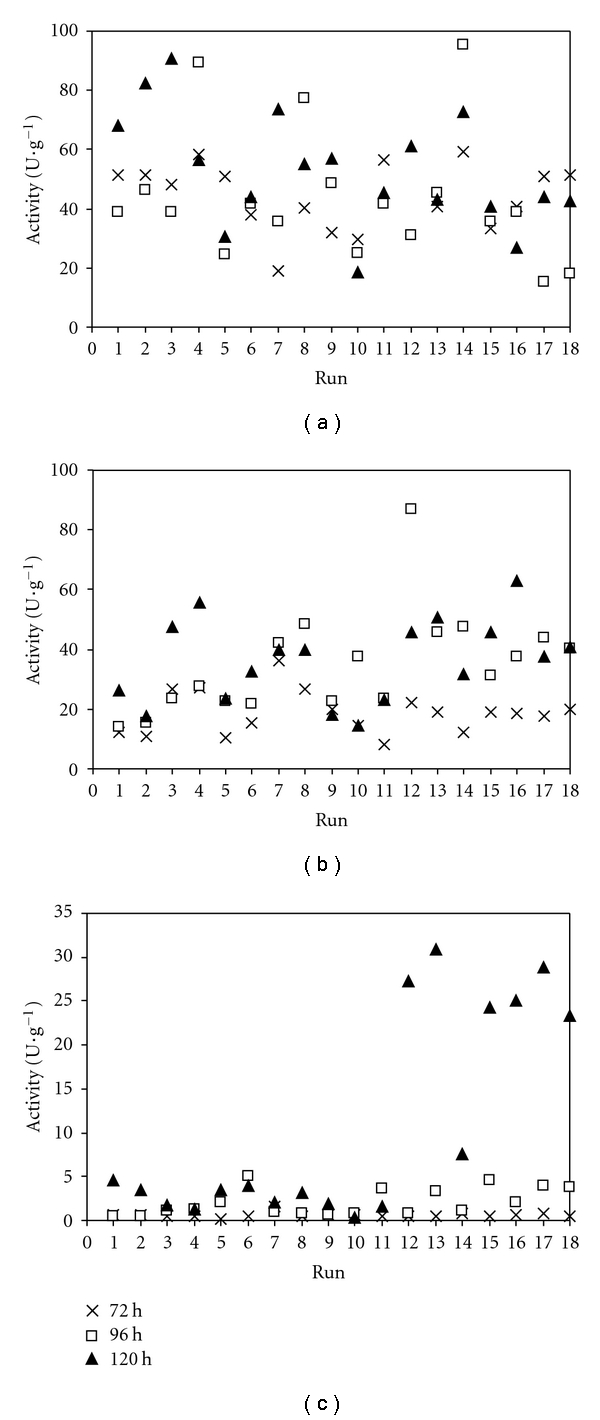
CCRD results for (a) endoamylases, (b) exoamylases, and (c) proteases production by *A. awamori* in SSF of babassu cake. Runs 15–18 correspond to central points.

The cells propagated in the liquid media with different C/N ratios for different times were then transferred to a solid medium composed of babassu cake, adjusting the initial moisture content to 70%. According to previous SSF experiments, fermentation time was studied in the range of 72–120 h. The activities of endoamylases, exoamylases, and proteases obtained for each experimental condition of the CCRD are presented in Figure 1. 

It can be observed that in general terms, in the media with high C/N ratios both the production of amylases and proteases were favored. In run 12, where initial C/N ratio, inoculum age, and concentration were highest, the maximum exoamylolytic and endoamylolytic activities and the second highest proteolytic activity were observed although in different times of SSF. Under these conditions, the maximum productivities for these three groups of enzymes, found after 72 h, 96 h, and 120 h of fermentation, were (1.49  ± 0.54) U g^−1^ h^−1^, (0.91  ± 0.02) U g^−1^ h^−1^ and (0.227  ± 0.004) U g^−1^ h^−1^, respectively. The observation that in run 12 endoamylases were produced prior to exoamylases is in accordance with the role of each of these enzyme groups in the hydrolysis of starch, that is, with endoenzymes contributing to a rapid depolymerization of the polysaccharides and higher oligosaccharides [4] and exoenzymes to the final release of glucose [3].

It should also be noticed that in the samples from runs 12, 13, and central point replicates, where the highest proteolytic activities were observed, amylolytic activities were not decreased during the onset of proteases activity. This indicates that possibly the proteases were preferentially attacking the proteins from the feedstock (which is desired) or that the amylases produced are glycosylated and thus more stable in the presence of proteases [34, 35].

### 3.3. Statistical Analysis of Amylases and Proteases Production in SSF of Babassu Cake

The experimental results for amylases and proteases shown in Figure 1 were analyzed in terms of their statistical significance, mainly by means of ANOVA and normality of residues, based on the tests of Kolmogorov-Smirnov and Lilliefors [36] and Shapiro and Wilk [37]. All nine data groups (three enzyme activities measured at 72, 96, and 120 h of SSF) showed adequate ANOVA and residues data, thus validating the statistical analyses.

Regression models were generated, considering the statistically significant terms, as well as those that were not significant, but that, when removed, would worsen model adjustment. Equations (4)–(6) show the adjusted models for the production of exoamylases (*Y*_1_), endoamylases (*Y*_2_) and proteases (*Y*_3_) by *A. awamori* IOC-3914 in babassu cake 

*72 h:*

(4) $$\begin{array}{cc} Y_{1} & =31.7851-\;3.4265X_{1}+0.0835X_{1}^{2}+1.5676X_{2}\\ & \;+0.8936X_{3},\\ Y_{2} & =24.5007+5.7361X_{1}-0.1354X_{1}^{2}-6.3310X_{2}\\ & \;+0.2490X_{2}^{2},\\ Y_{3} & =0.3876-0.0337X_{1}-0.0289X_{2}+0.2557X_{3}\\ & \;+0.0044X_{1}X_{2}-0.0043X_{1}X_{2}-0.0115X_{2}X_{3}, \end{array}$$

*96 h:*

(5) $$\begin{array}{cc} Y_{1} & =-62.3051+6.7032X_{1}-0.1481X_{1}^{2}+0.3990X_{2}\\ & \;+0.0651X_{1}X_{2},\\ Y_{2} & =133.5655-0.7615X_{1}-2.4532X_{2}-30.1873X_{3}\\ & \;+2.4476X_{3}^{2}+0.5541X_{2}X_{3},\\ Y_{3} & =-18.1781+1.1669X_{1}-0.0210X_{1}^{2}+0.7683X_{2}\\ & \;-0.0109X_{2}^{2}+0.9718X_{3}-0.0925X_{3}^{2}-0.0177X_{1}X_{2}\\ & \;+0.0190X_{1}X_{3}-0.0241X_{2}X_{3}, \end{array}$$

*120 h:*

(6) $$\begin{array}{cc} Y_{1} & =-107.615+9.7760X_{1}-0.1870X_{1}^{2}+5.1800X_{2}\\ & \;-0.059X_{2}^{2}-0.7500X_{3}-0.0940X_{1}X_{2},\\ Y_{2} & =58.4672-1.6609X_{1}+4.5235X_{2}-4.1081X_{3}\\ & \;+1.2008X_{3}^{2}-0.6322X_{2}X_{3},\\ Y_{3} & =-145.0540+10.2570X_{1}-0.2230X_{1}^{2}+4.298X_{2}\\ & \;-0.136X_{2}^{2}+8.508X_{3}-0.836X_{3}^{2}. \end{array}$$

For each fermentation time (72 h, 96 h, and 120 h), the models describing the production of the three groups of enzymes were analyzed jointly through the use of the global desirability function, *D* [38], which represents a geometric mean of the desirabilities of each response (*d*_*i*_). Since the experimental results (Figure 1) showed that the maximum activities of the different enzyme groups were achieved under distinct conditions, the multiresponse optimization was done in view of two scenarios: (1) considering only the production of exoamylases and endoamylases and (2) considering the production of all the three groups of enzymes. Results of these analyses are presented in Table 4. Higher *D* values were obtained in the first scenario, when only the amylolytic activities were taken into consideration. This is in agreement with the fact that the production of these enzymes was favored under more similar conditions than when proteases were also considered. The maximum *D* value possible is 1, and it would represent a perfect combination of the experimental conditions for the production of all enzymes.

**Figure 2:**
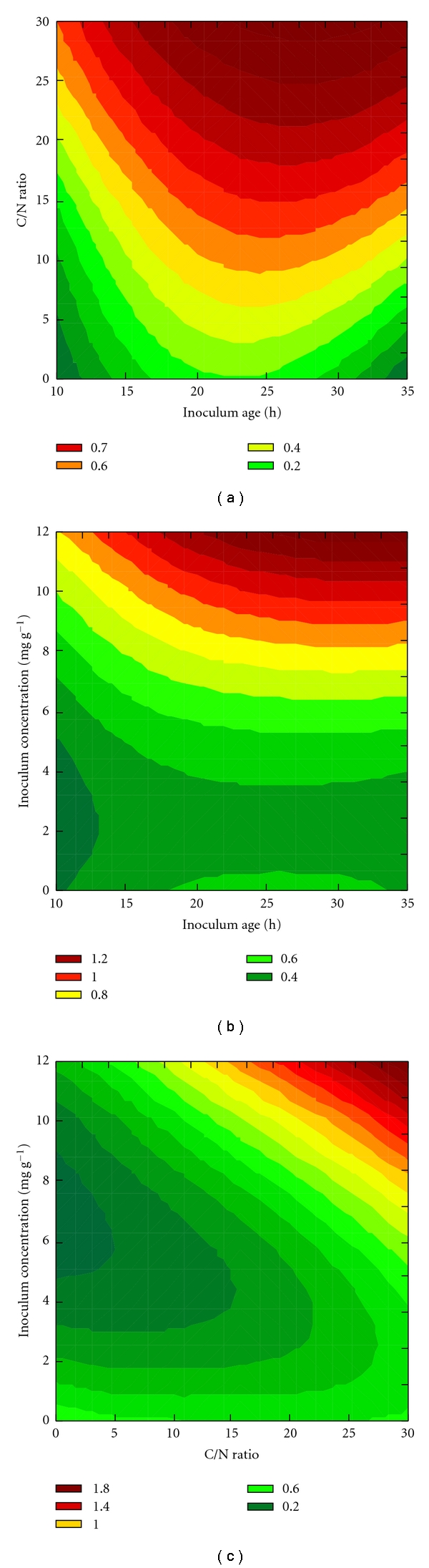
Response surfaces for the global desirability function (*D*) for simultaneous analysis of exoamylases and endoamylases after 96 h of fermentation of babassu cake by *A. awamori*.

Besides giving the highest *D* values, the simultaneous analysis of the production of exoamylases and endoamylases after 96 h of SSF predicted also the highest enzyme activities, thus indicating that optimizing the inoculum propagation conditions could effectively enhance the production of amylolytic enzymes. Thus, further analyses were concentrated on this fermentation time. The response surfaces of the desirability function (considering only the activities of amylases) for each pair of factors are presented in Figure 2. The surfaces indicate that the three factors exert a significant influence on the combination of the responses (represented by *D*) and that at least inoculum concentration and C/N ratio should be maximized to enhance the production of the amylolytic enzymes. This is in agreement with the results reported by Djekrif-Dakhmouche et al. [39], who studied the production of amylases by *A. niger*, concluding that the C/N ratio of the medium should be at least 20.

### 3.4. Experimental Validation of the Predicted Results

Experimental runs were carried out in order to validate if the best inoculum conditions (inoculum age of 28.4 h, C/N ratio of 25.8, and inoculum concentration of 9.1 mg g^−1^), as predicted by the desirability function analysis considering both amylolytic activities after 96 h of SSF, were really optimal. These runs were carried out in two scales: at the same scale as the CCRD runs and at a 4-fold larger scale. The results of replicates carried out at both scales are shown in Table 5. Considering a 95% confidence interval (1.96*SD) for both the predicted and the experimental results, the enzyme activities obtained experimentally did validate the optimum conditions determined by the desirability analysis.

The desirability function has been used for the multiresponse optimization of the production of enzymes, such as proteases and catalases [40] and cellulases [41] as well as for the application of enzymes, as reported by Castro et al. [29] regarding the use of multienzyme complexes containing amylases and proteases for the hydrolysis of babassu case.

## 4. Conclusions

Inoculum conditions for the simultaneous production of amylolytic and proteolytic enzymes were optimized using a multiresponse approach based on the desirability function. Kinetic profiles for the growth of *Aspergillus awamori* IOC-3914 in liquid medium containing malt extract and peptone were studied and used to select the variables that were subsequently investigated using design of experiments. Models were obtained to describe the production of each of the three enzyme groups under study (exoamylases, endoamylases and proteases) at different SSF process times (72, 96, and 120 h). A statistical analysis using the global desirability function indicated the inoculum conditions that would optimize enzyme production (inoculum age of 28.4 h, C/N ratio of 25.8, and inoculum concentration of 9.1 mg g^−1^), and these optimum conditions were validated experimentally, yielding exoamylases, endoamylases, and proteases activities of 55.4, 104.3, and 17.0 U g^−1^, respectively. The use of the desirability function showed to be a useful tool for the optimization of enzyme production.

## Figures and Tables

**Table 1:** CCRD matrix for optimization of inoculum conditions of *A. awamori* IOC-3914 for amylases and proteases production by SSF.

Run	Factor levels		
	Inoculum age (h)	Initial C/N ratio	Inoculum concentration (mg_biomass_ g^−1^_cake_)
1	16.0	7.6	3.5
2	16.0	7.6	8.2
3	16.0	21.2	3.5
4	16.0	21.2	8.2
5	30.0	7.6	3.5
6	30.0	7.6	8.2
7	30.0	21.2	3.5
8	30.0	21.2	8.2
9	11.2	14.4	5.8
10	34.8	14.4	5.8
11	23.0	3.0	5.8
12	23.0	25.8	5.8
13	23.0	14.4	1.9
14	23.0	14.4	9.7
15–18 (PC)	23.0	14.4	5.8

**Table 2:** Specific growth rates and doubling times, calculated according to the logistic model, presented by *A. awamori* when grown in culture media with different C/N ratios.

Initial C/N ratio	Specific growth rate (h^−1^)	Doubling time (h)	*R^2^*
3.0	0.015	46.3	0.919
7.6	0.031	22.4	0.774
14.4	0.063	11.0	0.851
21.2	0.112	6.2	0.846
25.8	0.152	4.6	0.798

**Table 3:** Biomass concentration after propagation of *A. awamori* in liquid culture media with different C/N ratios or different times.

Culture conditions		Biomass concentration (g L^−1^)
Initial C/N ratio	Inoculum age (h)	
3.0	23	1.16 ± 0.03
7.6	16	0.31 ± 0.02
7.6	30	4.50 ± 1.27
14.4	11	0.83 ± 0.08
14.4	23	7.58 ± 0.18
14.4	34	7.69 ± 1.32
21.2	16	1.44 ± 0.03
21.2	30	7.59 ± 1.13
25.8	23	7.80 ± 1.70

**Table 4:** Optimum inoculation conditions and enzyme activities predicted by means of the global desirability function, applied to two scenarios: considering only the amylolytic activities and considering also the proteases activity.

Predicted parameters	SSF time		
	72 h	96 h	120 h
Simultaneous analysis of endoamylase, and exoamylase production			

*D* value	0.439	0.902	0.763
Optimum inoculum age (h)	21.1	28.4	20.2
Optimum C/N ratio	25.8	25.8	25.8
Optimum inoculum concentration (mg g^−1^)	7.8	9.1	1.9
Exoamylolytic activity in optimum condition (U g^−1^)	27.1	73.6	52.5
Endoamylolytic activity in optimum condition (U g^−1^)	67.8	115.6	99.2

Simultaneous analysis of endoamylase, exoamylase, and proteases production			

*D* value	0.549	0.524	0.613
Optimum inoculum age (h)	33.7	25.8	21.8
Optimum C/N ratio	25.8	14.6	21.0
Optimum inoculum concentration (mg g^−1^)	3.5	9.7	2.8
Exoamylolytic activity in optimum condition (U g^−1^)	42.3	40.3	53.0
Endoamylolytic activity in optimum condition (U g^−1^)	45.4	92.0	66.1
Proteolytic activity in optimum condition (U g^−1^)	1.7	2.3	20.3

**Table 5:** Enzyme activities obtained in fermentations carried out for 96 h to validate the inoculum conditions predicted by desirability analysis to be optimal (inoculum age of 28.4 h, C/N ratio of 25.8, and inoculum concentration of 9.1 mg g^−1^).

Replicate	Activity (U g^−1^)		
	Exoamylase	Endoamylase	Protease
Smaller scale-1	45.0	135.4	7.6
Smaller scale-2	44.2	113.9	21.1
Smaller scale-3	53.4	90.3	19.8
Smaller scale-4	63.9	100.3	23.3
Smaller scale-5	59.5	71.7	13.1
Larger scale	60.7	119.3	17.3
Overall mean	55.4	104.3	17.0
Overall SD	7.6	24.1	5.5
Values predicted by means of the desirability function	73.6	115.6	—

## References

[1] Pérez S, Baldwin PM, Gallant DJ, BeMiller J, Whistler R (2009). Structural features of starch granules I. *Starch—Chemistry and Technology*.

[2] Bothast RJ, Schlicher MA (2005). Biotechnological processes for conversion of corn into ethanol. *Applied Microbiology and Biotechnology*.

[3] Norouzian D, Akbarzadeh A, Scharer JM, Young MM (2006). Fungal glucoamylases. *Biotechnology Advances*.

[4] van der Maarel MJEC, van der Veen B, Uitdehaag JCM, Leemhuis H, Dijkhuizen L (2002). Properties and applications of starch-converting enzymes of the *α*-amylase family. *Journal of Biotechnology*.

[5] Baruque Filho EA, Baruque MDGA, Sant’Anna GL (2000). Babassu coconut starch liquefaction: an industrial scale approach to improve conversion yield. *Bioresource Technology*.

[6] Melo WC, Dos Santos AS, Santa Anna LMM, Pereira N (2008). Acid and enzymatic hydrolysis of the residue from Castor Bean (*Ricinus communis* L.) oil extraction for ethanol production: detoxification and biodiesel process integration. *Journal of the Brazilian Chemical Society*.

[7] Nghiem NP, Hicks KB, Johnston DB (2010). Production of ethanol from winter barley by the EDGE (enhanced dry grind enzymatic) process. *Biotechnology for Biofuels*.

[8] Rao MB, Tanksale AM, Ghatge MS, Deshpande VV (1998). Molecular and biotechnological aspects of microbial proteases. *Microbiology and Molecular Biology Reviews*.

[9] Viniegra-González G, Favela-Torres E, Aguilar CN, Rómero-Gomez SDJ, Díaz-Godínez G, Augur C (2003). Advantages of fungal enzyme production in solid state over liquid fermentation systems. *Biochemical Engineering Journal*.

[10] dos Reis Castilho L, Polato CMS, Baruque EA, Sant’Anna GL, Freire DMG (2000). Economic analysis of lipase production by *Penicillium restrictum* in solid-state and submerged fermentations. *Biochemical Engineering Journal*.

[11] Hölker U, Lenz J (2005). Solid-state fermentation—are there any biotechnological advantages?. *Current Opinion in Microbiology*.

[12] Mitchell DA, Pandey A, Sangsurasak P, Krieger N (1999). Scale-up strategies for packed-bed bioreactors for solid-state fermentation. *Process Biochemistry*.

[13] Gutarra MLE, Godoy MG, dos Reis Castilho L, Freire DMG (2007). Inoculum strategies for Penicillium simplicissimum lipase production by solid-state fermentation using a residue from the babassu oil industry. *Journal of Chemical Technology and Biotechnology*.

[14] de Castro AM, Carvalho DF, Freire DMG, dos Reis Castilho L (2010). Economic analysis of the production of amylases and other hydrolases by *Aspergillus awamori* in solid-state fermentation of babassu cake. *Enzyme Research*.

[15] Kammoun R, Naili B, Bejar S (2008). Application of a statistical design to the optimization of parameters and culture medium for *α*-amylase production by *Aspergillus oryzae* CBS 819.72 grown on gruel (wheat grinding by-product). *Bioresource Technology*.

[16] Prakasham RS, Subba Rao C, Sreenivas Rao R, Sarma PN (2007). Enhancement of acid amylase production by an isolated *Aspergillus awamori*. *Journal of Applied Microbiology*.

[17] Sindhu R, Suprabha GN, Shashidhar S (2009). Optimization of process parameters for the production of *α*-amylase from *Penicillium janthinellum* (NCIM 4960) under solid state fermentation. *African Journal of Microbiology Research*.

[18] Esfahanibolandbalaie Z, Rostami K, Mirdamadi SS (2008). Some studies of *α*-amylase production using *Aspergillus oryzae*. *Pakistan Journal of Biological Sciences*.

[19] Kunamneni A, Permaul K, Singh S (2005). Amylase production in solid state fermentation by the thermophilic fungus Thermomyces lanuginosus. *Journal of Bioscience and Bioengineering*.

[20] Nahas E, Waldemarin MM (2002). Control of amylase production and growth characteristics of *Aspergillus ochraceus*. *Revista Latinoamericana de Microbiología*.

[21] Ramachandran S, Patel AK, Nampoothiri KM (2004). Alpha amylase from a fungal culture grown on oil cakes and its properties. *Brazilian Archives of Biology and Technology*.

[22] Ramachandran S, Patel AK, Nampoothiri KM (2004). Coconut oil cake—a potential raw material for the production of *α*-amylase. *Bioresource Technology*.

[23] Farooq S, Iqbal SM, Rauf CA (2005). Physiological studies of *Fusarium oxysporum* F. Sp. Ciceri. *International Journal of Agriculture and Biology*.

[24] Bhanja T, Rout S, Banerjee R, Bhattacharyya BC (2007). Comparative profiles of *α*-amylase production in conventional tray reactor and GROWTEK bioreactor. *Bioprocess and Biosystems Engineering*.

[25] Kathiresan K, Manivannan S (2006). *α*-amylase production by *Penicillium fellutanum* isolated from mangrove rhizosphere soil. *African Journal of Biotechnology*.

[26] Murthy PS, Naidu MM, Srinivas P (2009). Production of *α*-amylase under solid-state fermentation utilizing coffee waste. *Journal of Chemical Technology and Biotechnology*.

[27] Sivaramkrishnan S, Gangadharan D, Nampoothiri KM, Soccol CR, Pandey A (2007). Alpha amylase production by *Aspergillus oryzae* employing solid-state fermentation. *Journal of Scientific and Industrial Research*.

[28] Mitchell DA, von Meien OF, Krieger N, Dalsenter FDH (2004). A review of recent developments in modeling of microbial growth kinetics and intraparticle phenomena in solid-state fermentation. *Biochemical Engineering Journal*.

[29] de Castro AM, de Andréa TV, dos Reis Castilho L, Freire DMG (2010). Use of mesophilic fungal amylases produced by solid-state fermentation in the cold hydrolysis of raw babassu cake starch. *Applied Biochemistry and Biotechnology*.

[30] Calado V, Montgomery D (2003). *Planejamento de Experimentos Usando o Statistica*.

[31] Hellendoorn L, Mulder H, Van den Heuvel JC, Ottengraf SPP (1998). Intrinsic kinetic parameters of the pellet forming fungus *Aspergillus awamori*. *Biotechnology and Bioengineering*.

[32] Barbosa AM, Cunha PDT, Pigatto MM, Silva MLC (2004). Produção e aplicação de exopolissacarídeos fúngicos. *Semina: Ciências Exatas e Tecnológicas*.

[33] Gutarra MLE, De Godoy MG, Silva JDN (2009). Lipase production and *Penicillium simplicissimum* morphology in solid-state and submerged fermentations. *Biotechnology Journal*.

[34] James JA, Lee BH (1997). Glucoamylases: microbial sources, industrial applications and molecular biology—a review. *Journal of Food Biochemistry*.

[35] Moreira FG, Lenartovicz V, Peralta RM (2004). A thermostable maltose-tolerant *α*-amylase from *Aspergillus tamarii*. *Journal of Basic Microbiology*.

[36] Lilliefors HW (1967). On the Kolmogorov-Smirnov test for normality with mean and variance unknown. *Journal of the American Statisical Association*.

[37] Shapiro SS, Wilk MB (1965). An analysis of variance test for normality (complete samples). *Biometrika*.

[38] Derringer G, Suich R (1980). Simultaneous optimization of several response variables. *Journal of Quality Technology*.

[39] Djekrif-Dakhmouche S, Gheribi-Aoulmi Z, Meraihi Z, Bennamoun L (2006). Application of a statistical design to the optimization of culture medium for *α*-amylase production by *Aspergillus niger* ATCC 16404 grown on orange waste powder. *Journal of Food Engineering*.

[40] Mandal C, Gudi RD, Suraishkumar GK (2005). Multi-objective optimization in *Aspergillus niger* fermentation for selective product enhancement. *Bioprocess and Biosystems Engineering*.

[41] Maeda RN, Da Silva MMP, Santa Anna LMM, Pereira N (2010). Nitrogen source optimization for cellulase production by *Penicillium funiculosum*, using a sequential experimental design methodology and the desirability function. *Applied Biochemistry and Biotechnology*.

